# Leydig Cells in Patients with Non-Obstructive Azoospermia: Do They Really Proliferate?

**DOI:** 10.3390/life11111266

**Published:** 2021-11-19

**Authors:** Dinko Hauptman, Marta Himelreich Perić, Tihana Marić, Ana Katušić Bojanac, Nino Sinčić, Zoran Zimak, Željko Kaštelan, Davor Ježek

**Affiliations:** 1 Scientific Centre of Excellence for Reproductive and Regenerative Medicine, School of Medicine, University of Zagreb, 10000 Zagreb, Croatia; dinko.hauptman@kbc-zagreb.hr (D.H.); marta.himelreich@mef.hr (M.H.P.); tihana.maric@mef.hr (T.M.); ana.katusic@mef.hr (A.K.B.); nino.sincic@mef.hr (N.S.); zoran.zimak@kbc-zagreb.hr (Z.Z.); zeljko.kastelan@kbc-zagreb.hr (Ž.K.); 2 Department of Urology, University Hospital Zagreb, 10000 Zagreb, Croatia; 3 Department of Medical Biology, School of Medicine, University of Zagreb, 10000 Zagreb, Croatia; 4 Department of Histology and Embryology, School of Medicine, University of Zagreb, 10000 Zagreb, Croatia; 5 Department of Transfusion Medicine and Transplantation Biology, University Hospital Zagreb, 10000 Zagreb, Croatia

**Keywords:** azoospermia, testicular biopsy, Leydig cells, cell hypertrophy, cell hyperplasia, morphometry, stereology

## Abstract

Background: Non-obstructive azoospermia (NOA) is a form of male infertility caused by disorders of the testicular parenchyma and impaired spermatogenesis. This study aimed to investigate the nature of Leydig cell changes in patients with NOA, especially whether their actual proliferation occurred. Methods: 48 testicular biopsies from infertile patients with NOA and 24 testicular biopsies originating from azoospermic patients suffering from obstructive azoospermia (OA) were included in the study. Leydig cells and their possible proliferative activity were analysed by immunohistochemistry and morphometry (stereology). Results: Unlike in the OA group, Leydig cells in NOA patients were sometimes organised into larger clusters and displayed an abundant cytoplasm/hypertrophy. Moreover, significant fibrosis of the interstitial compartment was demonstrated in some NOA samples, often accompanied by inflammatory cells. Stereological analysis showed no increase/proliferation of Leydig cells; on the contrary, these cells decreased in number in the NOA group. Conclusions: The decrease in the number of Leydig cells can be explained by previous inflammatory changes within the testicular interstitium and consequent interstitial fibrosis. The interstitial fibrosis might have a deteriorating effect on Leydig cells.

## 1. Introduction

The most severe form of infertility is the complete absence of spermatozoa in a man’s semen, defined as azoospermia [1,2]. Azoospermia occurs in 1% of cases in the general population, while in the infertile male population, it occurs in 10–20% of cases. There are two forms of azoospermia: obstructive (OA) and non-obstructive azoospermia (NOA) [1]. OA is characterised by full perseverance of testicular parenchyma, including complete spermatogenesis [1,3]. In contrast, NOA cases display in histological findings various degrees of testicular structural damage. In 70–90% of cases, the aetiology of the disorder is unknown. The most common histological picture is mixed atrophy of seminiferous tubules; however, the damage of spermatogenesis could vary from a mild form (hypospermatogenesis) until severe one (maturation arrest, Sertoli cells only syndrome or tubular sclerosis) [3,4,5].

Studies on the function of seminiferous tubules of infertile men have shown an interdependence between the testicular tubular and interstitial compartment-part of the testis where Leydig cells are located. It is believed that disruption of the seminiferous epithelium within the tubules can cause changes in these cells (and vice-versa). Specifically, 12 to 15% of men with impaired spermatogenesis have lower testosterone levels and/or elevated follicle stimulating (FSH) and luteinizing hormone (LH) levels. Sometimes testosterone levels are at the lower limit of the normal range; thus, it is assumed that Leydig cells, despite the damage, could make up for the amount of testosterone that was missing [6,7,8]. Patients with idiopathic azoospermia in a significant number of cases (96%) have normal serum testosterone levels. Exceptionally, testosterone levels may also be lowered or slightly elevated [2,3,4,6,7,9]. However, these are often values close to the lower limit of the reference values with significantly increased LH, FSH and decreased values of inhibin B [3,5,6,7]. For example, patients receiving chemotherapy for lymphoma (and developing azoospermia) had low serum inhibin B, testosterone levels, and elevated LH. The ratio of testosterone to LH levels may well serve to assess the degree of Leydig cell damage. In idiopathic azoospermia, significantly elevated LH and testosterone at the lower limit of normal indicate functional “failure” of Leydig cells. It is estimated that 1 in 20 such patients will have severe consequences due to decreased testosterone production [10].

Measurement of estradiol (E2) in infertile patients revealed a subgroup of patients with significantly increased levels of this hormone in the blood. The relationship between E2 and testosterone is also disrupted [8]. Elevated E2 levels can cause a disturbance in testosterone synthesis (autocrine block enzyme 17 alpha-hydroxylase) in the cytoplasm of Leydig cells. It is also known that the source of estrogen in the seminiferous tubules is the Sertoli cell. It possesses receptors for FSH. This hormone is markedly elevated in patients with NOA. It is assumed that hyperstimulation of Sertoli cells with FSH can lead to increased estrogen production and thus to Leydig cell dysfunction. It remains unclear how hyperstimulation of Sertoli cells with FSH can damage Leydig cells. One possibility is, undoubtedly, the excessive amount of estrogen secreted by these cells [11,12]. Furthermore, recent studies in mice with intentional/targeted genetic modification (knock-out mice) have shown that FSH receptor deficiency is associated with infertility. Still, the same has not been demonstrated in animals with LH receptor deficiency [10,12].

In NOA patients, interstitial Leydig cells are thought to show signs of hypertrophy and/or hyperplasia due to persistently elevated gonadotropin levels in these patients [2,3,4,5]. Few studies have been conducted to confirm whether Leydig cells in patients with NOA undergo hypertrophy and hyperplasia [6]. Up to our knowledge, only one study, using morphometric methods, showed that Leydig cells exclusively hypertrophied. Still, the number of patients with NOA in the study was only 7, which could not confirm or rule out hyperplasia [13]. Bearing in mind the scarcity of data on Leydig cells hypertrophy/hyperplasia, our study aimed to give a detailed insight into the morphological changes of these cells in NOA patients. In particular, using immunohistochemistry and morphometry (stereology), we wanted to check if Leydig cells proliferate in azoospermic men with NOA. Moreover, based on our previous morphological observations on testicular biopsies, we wanted to investigate the possible link between the testis inflammation, interstitial fibrosis, and Leydig cells changes in infertile men.

## 2. Materials and Methods

### 2.1. Patients, Testicular Sampling, and Tissue Processing

All patients enrolled in the study were referred with a diagnosis of azoospermia to the Department of Urology, University Hospital Zagreb. Infertile men were of Croatian origin, treated through the National Health Service. Within 2013–2020, 309 azoospermic patients were submitted to a thorough urological work-up, including personal and family history, physical examination, semen analysis (at least two samples), hormone analysis, genetic testing (karyotype, Y-chromosome microdeletions, cystic fibrosis transmembrane conductance regulator gene /CFTR/ testing), ultrasound, and other relevant examinations. Out of 309 initially recruited patients, a total of 72 men diagnosed with idiopathic azoospermia was included in the study, of which 48 were diagnosed with NOA. The control group comprised 24 patients suffering from obstructive azoospermia (OA) with fully preserved testicular parenchyma. OA group consisted of men with an average age of 33.5 y. (age span 24–27 y.), whereas NOA group included patients with an average age of 32.8 y. (age span 22–43 y.). The distinction between OA and NOA was based upon testis volume, hormone profiles, and histology of the testicular biopsy. The study has been conducted in cooperation with the School of Medicine, University of Zagreb, and the approval for the research was given by the Ethics Committee of the School.

Testicular samples were obtained by the open testicular biopsy, which was described in the papers of Holstein et al. [14,15]. Tissue pieces were fixed in Bouin’s fixative immediately after excision. The usual histological procedure of tissue dehydration, fitting into paraffin blocks, cutting and staining with hematoxylin and eosin (H&E) was performed. Paraffin blocks were cut with a rotary microtome Leitz 1512 (Austria), where the thickness of the sections was 4 µm. Apart from staining with H&E and qualitative histological analysis (assessing the preservation of seminal epithelium and interstitium), the obtained serial sections were used for immunohistochemical analysis.

### 2.2. Immunohistochemistry

For immunohistochemical analysis, part of the serial paraffin sections of biopsies obtained from patients with OA and NOA was placed on silanised slides (S3003; DAKO, Glostrup, Denmark). The slides were allowed to air dry for 24 h in a thermostat (Tehnika, Ljubljana, Slovenia) at 37 °C. Cell proliferation was detected using rabbit monoclonal antibody to Ki-67 protein (MA5-14520, Thermo Fischer Scientific, Waltham, MA, USA, dilution 1:100) and proliferating cell nuclear antigen (PCNA, DAKO, dilution 1:100). The presence of Leydig cells was confirmed with two antibodies: against the antigen INSL3 (HPA028615, Sigma Aldrich, Taufkirchen, Germany, dilution 1:100) and testosterone (NBP2-45187, Novus Biologicals, Littleton, CO, USA, dilution 1:20). An indirect EnVision™ two-step staining method [16] was used to detect bound primary antibodies to the corresponding antigen [16] according to the protocol of the manufacturer (DAKO, Abcam, respectively). Before applying primary and secondary antibodies, the sections were circled with a grease pen (S2002; DAKO, Glostrup, Denmark).

To block the non-specific binding of the secondary antibody, a calf serum (5%, dissolved in Tris Buffered Saline, TBS) was applied on the sections and left for 20 min. The excess of the serum was then removed. Primary antibodies were pre-diluted in 1% Bovine Serum Albumin, BSA/TBS/0.05% Tween 20 (Sigma Aldrich, Taufkirchen, Germany). Sections were incubated with primary antibodies in a humid chamber overnight at +4 ºC. One section of each series was incubated with dilution buffer only (1% BSA/TBS/0.05% Tween 20) and served as a negative control [16,17,18,19]. Depending on the species specificity (mouse or rabbit), goat anti-rabbit IgG specific antibody (goat anti-rabbit Igg, 90751, Abcam, Cambridge, United Kingdom) or mouse immunoglobulin specific antibody (donkey anti-mouse Igg, ab182022, Abcam) were used (BSA/TBS in a ratio of 1:1000). The secondary antibody was applied for 1 h (goat) or 45 min (donkey), after which the sections were washed 1 × 5 min in TBS buffer.

The signal, i.e., the presence of bound antibody, was shown by incubation of sections with a solution of chromogen DAB + Chromogen (3,3′-diaminobenzidine) and peroxidase substrate (K3468, DAKO/Agilent, Glostrup, Denmark) for 3 min. The reaction was quenched by immersing the slides in distilled water two times. The sections were further contrasted by immersion in hematoxylin solution for 2 min and washed in cold tap water for 20 min until bluish. After rinsing in distilled water, the sections were dehydrated by immersion in increasing ethanol concentrations (70 to 100%, 5 min each). An incubation in xylene solution (3 × 5 min) followed. The slides were then covered with a fitting medium (DPX, 06522, Sigma) and allowed to dry for 24 h.

### 2.3. Morphometric (Stereological) Analysis

An initial (pilot) stereological measurement was performed in order to yield reliable data. Separate measurements were done for OA and NOA groups. The measurements included randomly chosen patients, randomly chosen blocks of paraffin-embedded bioptic material, and slides/sections. Morphometric analysis was performed on 4 µm thick paraffin sections. For stereological measurement, from 60 serial sections obtained from each biopsy sample, the first 10 and last 10 sections were selected. Thus, the physical dissector analysis was applied to avoid counting the same structures/cells [20,21]. Measurements were performed by a multifunctional Weibel test system with 42 test points inserted into the ocular. A Nikon Alphaphot binocular microscope (Vienna, Austria) with a total magnification of ×400 was used for all measurements. The area of the test system (At) at the stated magnification was 0.0837 mm^2^, the length of the test line (d) was 0.048 mm, and the total length of the test lines (Lt) was 1.008 mm.

During the stereological processing, the following stereological variables were determined:

NvLc-number of immunohistochemically positive Leydig cells on insulin-like hormone 3 (INSL 3) and testosterone (T) in a testicle unit volume (mm^3^ of tissue) (OA biopsies and testicle samples of patients with NOA);
NLc-total number of immunohistochemically positive Leydig cells on insulin-like hormone 3 (INSL 3) and testosterone (T) in the whole testis (OA biopsies and testis samples of patients with NOA).

The total number of immunohistochemically positive Leydig cells in the whole testis (NLc) (OA biopsies and testicle samples of patients with NOA) was calculated using the following formula [20,21,22,23]:

NLc = NLc/Ni × Vo(1)

NvLc = numerical density of immunohistochemically positive Leydig cells; Ni = numerical cell density in the testis interstitium; Vo = testicle volume).

Data on testicular volume determined by ultrasound were obtained from patients’ medical history at the Department of Urology, University Hospital Zagreb. 

To calculate the volume density of Leydig cells (VvLc), which indicates the portion of space occupied by these cells within the unit volume of testis (mm^3^), the following formula was used [20,21,22,23]:

VvLc = Pf/Pt(2)

VvLc = volume density of Leydig cells; Pf = Pf: the number of test points that fall into the area of seminiferous tubules; If = the number of intersections of the borderline and the lines within the test system) 

To calculate the total volume of Leydig cells (VLc) in the testis, the following formula was used:

VLc = VvLc × Vo(3)

The number of stereological measurements or number of areas (n) that had to be morphometrically analysed and to determine 95% confidence intervals, an orientational (pilot) measurement was done by measuring each variable in 5–10 test areas. Then, the arithmetic mean (x) and the standard deviation (s) were calculated, and these values were put into the following equation [20,21,22,23]:

*n* = (20 × s/x)^2^ (4)

The number of stereological measurements or number of areas (*n*) that had to be morphometrically analysed was 57 per section.

### 2.4. Statistical Analysis

Data analysis was performed at the Department of Medical Statistics, Epidemiology, and Medical Informatics, School of Public Health “Andrija Štampar”, School of Medicine University of Zagreb. All obtained data were entered into a computer and processed by a biostatistical program. STATISTICA version 12.0, (Palo Alto, CA, USA) (www.statsoft.com, accssesed on 14 May 2021) was used for the data processing. After computer data entry, whether the data (for the OA and infertile group of patients with NOA) followed the normal distribution was checked. Appropriate statistical methods of comparison of two independent groups of samples were applied. Based on the pilot stereological study, a study power analysis was performed for the OA and NOA groups. The analysis indicated that in order to obtain reliable data, the OA group should have included minimum 18 and NOA 43 cases.

The analysis of the normality of the distribution of numerical data (Smirnov-Kolmogorov test) was made. In accordance with the obtained results, appropriate parametric and/or non-parametric statistical analyses, and data display methods were applied. Quantitative data were presented through ranges, arithmetic means, and standard deviations, i.e., median and interquartile ranges in cases of non-parametric distribution. Differences in quantitative values between individual groups (NOA in relation to OA) were assessed by an independent t-test, i.e., the Mann–Whitney U test. Differences in variables between the examined groups were analysed by Fisher’s exact test, and Fisher-Freeman-Halton exact test in the case of contingency tables larger than 2 × 2 format. The corresponding correlation coefficients between the findings of hypertrophy and hyperplasia of Leydig cells and other variables were calculated. All *p* values less than 0.05 were considered significant.

## 3. Results

### 3.1. Hormone Values

Main hormone parameters in investigated groups of patients are presented in Table 1. The OA group had normal values of follicle stimulating hormone (FSH), luteinizing hormone (LH) and total testosterone (T). Patients in the NOA group displayed significantly increased values of FSH and LH, including one case where the value of FSH was 41.9 UI/L. As for T values in the NOA group, 41 patients had normal values; 5 patients had decreased values, whereas 2 patients had increased T values (Table 1).

### 3.2. Results of the Qualitative Histological Analysis

#### 3.2.1. Qualitative Histological Analysis of the Control Group of Biopsies

Histological analysis on sections stained with H&E of the OA group showed a high degree of preservation of the testicular parenchyma in all biopsy samples. Biopsy fragments averaged 40–50 seminiferous tubules with the associated interstitium. The diameter of the tubules was 170–190 µm, with the regular stratified seminiferous epithelium. The epithelium showed all stages of fully developed spermatogenesis and was resting on a thin basement membrane beneath which laid the lamina propria. The lamina propria consisted of 5–7 layers of elongated peritubular (myoid) cells. The seminiferous epithelium contained supporting Sertoli cells and all forms of spermatogenic cells: spermatogonia, primary and secondary spermatocytes, immature and mature spermatids and spermatozoa.

**Figure 1:**
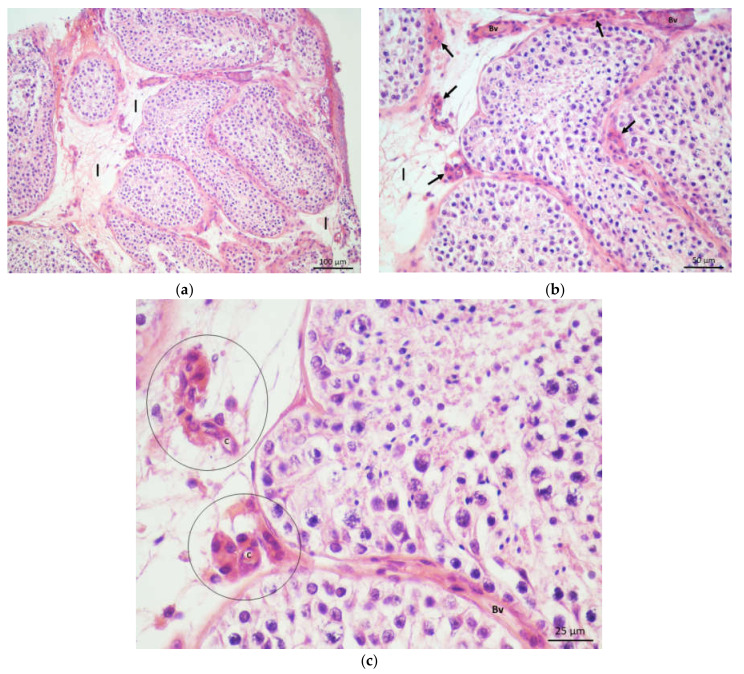
(**a**) Testicular biopsy from a patient with obstructive azoospermia. Seminiferous tubules displayed normal diameter and were lined with seminiferous epithelium. Between the tubules was the interstitial loose connective tissue (I). (H&E, ×100, scale bar = 100 μm); (**b**) Detail from Figure 1a. Seminiferous epithelium retained complete spermatogenesis. Within the interstitium (I), regular clusters of Leydig cells (→) and blood vessels (Bv) were visible. (H&E, ×200, scale bar = 50 μm). (**c**) Normal clusters of Leydig cells (circled) were nourished by a fine network of capillaries (**c**) (Bv, blood vessel) (H&E, ×400, scale bar = 25 μm).

Between seminiferous tubules was a loose interstitial connective tissue. Interstitial Leydig cells were seen in smaller groups (3–15 cells). These cells were noticeably regular in appearance, mostly polygonal. The round or oval nucleus of loose chromatin often contained a well-visible nucleolus. The cytoplasm was abundant, with a few drops of fat vacuoles. Some Leydig cells were arranged around blood vessels located in the central parts of the interstitial spaces (so-called perivascular Leydig cells). Leydig cells in the immediate vicinity of the seminiferous tubules were elongated and often followed the appearance of their cytoplasm by the outer contour of the tubules (so-called peritubular Leydig cells). As already mentioned, smaller or larger blood vessels could be observed near Leydig cells, including an abundant network of blood capillaries. All connective tissue cell types were also found, mostly fibrocytes and fibroblasts (Figure 1a–c).

#### 3.2.2. Qualitative Histological Analysis of Testicular Biopsies Obtained from NOA Patients

**Figure 2:**
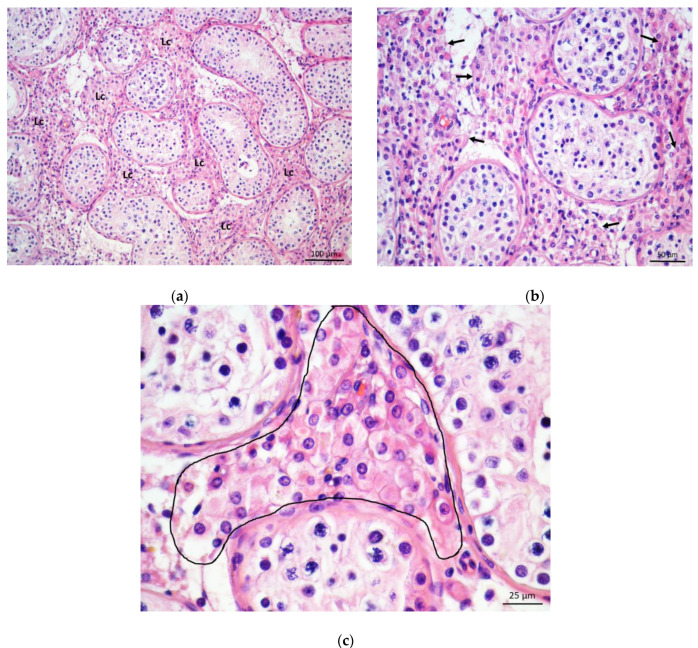
(**a**) Testicular parenchyma obtained from a patient with a maturation arrest (spermatid “stop”). Seminiferous tubules had slightly decreased diameter. In the interstitial space, large groups of Leydig cells (Lc) were visible. (H&E, ×100, scale bar = 100 μm); (**b**) Detail of testicular biopsy from the previous picture. Spermatogenesis ran until the round spermatid stage. Mature spermatids and spermatozoa were lacking. Leydig cells (→) show hypertrophic changes. (H&E, ×200, scale bar = 50 μm). (**c**) One of Leydig cells clusters (encircled), high magnification. Conspicuous nuclei of Leydig cells were surrounded by an abundant cytoplasm. (H&E, ×400, scale bar = 25 μm).

In biopsy specimens of patients with NOA, different degrees of damage of spermatogenesis were noted. In three cases, a maturation arrest of spermatogenic cells could be observed. In the case of a spermatid “stop”, the seminiferous epithelium was rich in immature (round) spermatids, whereas mature (elongated) spermatids and spermatozoa were missing (Figure 2a–c).

**Figure 3:**
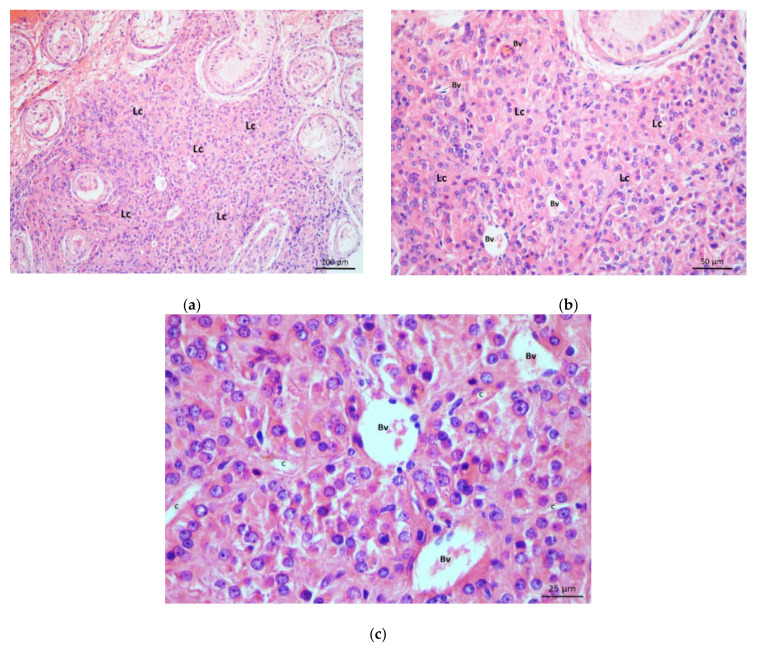
(**a**) Part of the testicular biopsy from a patient with Sertoli cells only syndrome. Seminiferous tubules were lined with Sertoli cells exclusively. A massive cluster of Leydig cells (Lc) was located between the tubules. (H&E, ×100, scale bar = 100 μm); (**b**) Detail from Figure 3a. Leydig cells (Lc) had enlarged cytoplasm. The cluster was supplied by a rich network of blood vessels (Bv). Part of one seminiferous tubule was lined only with columnar Sertoli cells. (H&E, ×200, scale bar = 50 μm). (**c**) Within the cluster, Leydig cells demonstrated large nuclei with one or several nucleoli and well-developed cytoplasm. (Bv, blood vessels; c, capillaries) (H&E, ×400, scale bar = 25 μm).

In more severe cases, spermatogenic cells were reduced to primary spermatocytes (“spermatocyte stop”) or spermatogonia (“spermatogonia only”). Moreover, 11 NOA cases presented with the picture of Sertoli cells only syndrome, where seminiferous tubules were devoid of spermatogenic cells, and only somatic (Sertoli) cells remained in the tubules (Figure 3a,b). In some NOA cases, apart from tubules lined only with Sertoli cells, fibrotic tubules were found. In those biopsies, seminiferous tubules were transformed into strands of fibrotic tissue, often referred to as “tubular shadows”. However, in the vast majority of NOA cases (34 or 71%), seminiferous tubules displayed a “mixed atrophy” pattern, i.e., combination of histology pictures described above.

**Figure 4:**
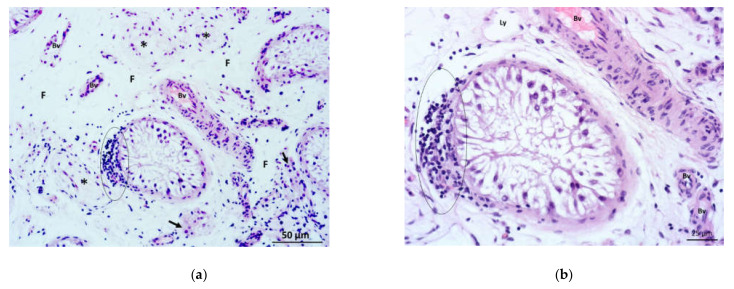
(**a**) Testicular parenchyma from the patient with Sertoli cells only syndrome and interstitial fibrosis. Seminiferous tubules had a sharply decreased diameter. There was no spermatogenesis going on. Some tubules were sclerotic (*) and transformed into strands of connective tissue called “tubular shadows”. Between the tubules, there were sizeable fibrotic areas (F) devoid of Leydig cells. Some reduced clusters of Leydig cells (→) were found distant to fibrotic regions. The peritubular area of one seminiferous tubule was infiltrated with mononuclear cells (circled area). (Bv, blood vessels). (H&E, ×200, scale bar = 50 μm); (**b**) Detail from Figure 4a. Mononuclear cells (circled area) infiltrated the peritubular region as well as the lamina propria of a seminiferous tubule lined with Sertoli cells. (Bv, blood vessels; Ly, lymph vessel) (H&E, ×400, scale bar = 25 μm).

In the testicular biopsy samples of patients with NOA, the interstitium contained Leydig cells of diverse morphology. In many parts of the given bioptic specimen, Leydig cells retained their characteristic appearance. They were arranged in smaller groups of 3–15 cells, close to blood vessels and/or seminiferous tubules (perivascular and peritubular Leydig cells). These cells were easily recognisable by their abundant eosinophilic cytoplasm, a well-visible nucleus with loose chromatin and sometimes prominent nucleolus. However, in many NOA cases, larger clusters of Leydig cells (30–80), which occupied a significant proportion of interstitial space, were often found (even in the same biopsy) (Figure 2a–c and Figure 3a–c). There were also Leydig cells with more abundant cytoplasm and signs of overt hypertrophy within these larger clusters. In the groups of such hypertrophic Leydig cells, rich blood vessels, especially delicate capillaries, could be seen (Figure 2b,c and Figure 3b,c). Despite abundant groups of Leydig cells described above, no mitotic figures were observed in H&E sections. However, assessing the interstitial tissue of some NOA patients, the opposite phenomenon could be observed. Certain parts of the interstitium of the testes of patients with NOA were intensely fibrosed, with an abundant presence of fibroblasts and fibrocytes in the intercellular space. It was apparent interstitial fibrosis with few Leydig cells remaining in the interstitium. These cells had elongated cytoplasm and were surrounded by rich connective tissue (Figure 4a,b).

**Figure 5:**
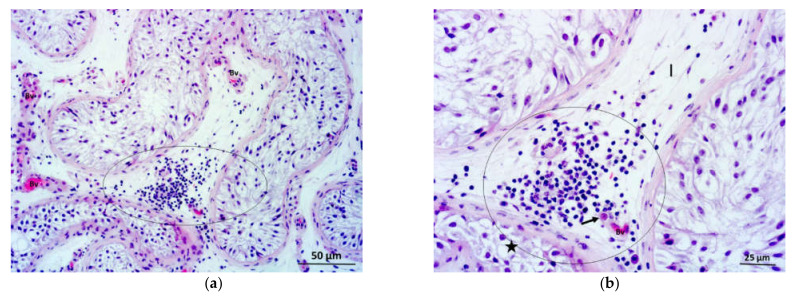
(**a**) Testicular biopsy from a patient with Sertoli cells only syndrome and accompanying inflammation. Seminiferous tubules had a narrow lumen and were devoid of any spermatogenic cells. In one part of the interstitium between the neighbouring tubules, the loose connective tissue was infiltrated by mononuclear cells (circled area). (Bv, blood vessels) (H&E, ×200, scale bar = 50 μm); (**b**) Detail of the previous picture. Mononuclear cells (circled area) were found inside the interstitium (I) and in close contact with Leydig cell (→) and blood vessel (Bv). Nearby seminiferous epithelium consisted of Sertoli cells only. (H&E, ×400, scale bar = 25 μm).

In several biopsy samples from patients with NOA, focal infiltrates of inflammatory cells-lymphocytes and monocytes/macrophages (mononuclear cells) were observed. These infiltrates were located primarily close to individual seminiferous tubules (Figure 4a,b), permeating the surrounding loose connective tissue of the tubules, peritubular Leydig cells, and part of the lamina propria of the tubules. At the same time, significant areas of the adjacent interstitium were fibrosed (Figure 4a). In some biopsy specimens, diffuse infiltrates of the same inflammatory cells were observed in the loose interstitial connective tissue (most commonly around smaller blood vessels) (Figure 5a). The inflammatory cells were often located close to perivascular Leydig cells (Figure 5b). Finally, a combination of mononuclear infiltrates was observed, which were located both around the seminiferous tubules as well as in the loose interstitial connective tissue.

### 3.3. Results of Immunohistochemistry Analysis

**Figure 6:**
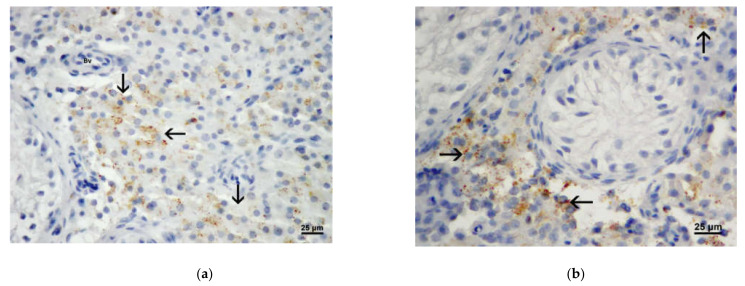
(**a**) A part of the testicular biopsy obtained from a man with OA. Testosterone expression (→) in Leydig cells showed a fine granular pattern (Bv, blood vessel) (DAB, ×400, scale = 25 µm); (**b**) Testosterone expression (→) in a testicular biopsy from a patient with Sertoli cells only syndrome. Solely Leydig cells were positive. Seminiferous tubule was lacking spermatogenic cells. (DAB, ×400, scale = 25 µm).

**Figure 7:**
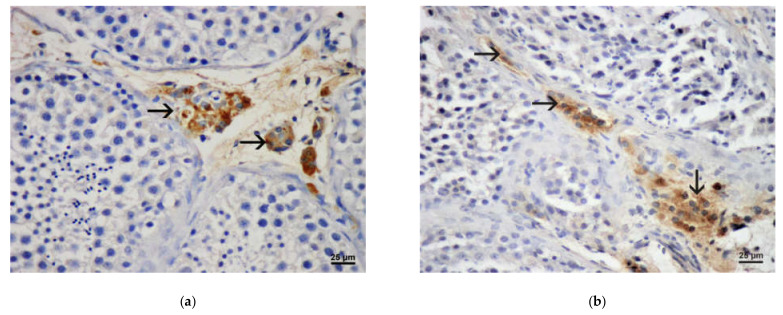
(**a**) Expression of INSL3 (→) in Leydig cells of the biopsy obtained from a patient with OA.). (DAB, ×400, scale = 25 µm) (**b**) Expression of INSL3 (→) in Leydig cells of a patient with NOA (hypospermatogenesis). (DAB, ×400, scale = 25 µm).

In addition to morphological features of Leydig cells, antibodies to testosterone and INSL3 (insulin-like factor 3) were used to detect Leydig cells in the testis interstitium. Testosterone was expressed in Leydig cells under the current influence of hypothalamic and pituitary hormones, and INSL3 was found to be a characteristic marker of fully differentiated Leydig cells. Numerous Leydig cells (in the bioptic material of both OA and NOA patients) with a positive signal were found during immunohistochemical staining with an antibody to testosterone (Figure 6a,b). The signal obtained with antibody against INSL3 proved to be an even more sensitive Leydig cells marker (Figure 7a,b). With both antibodies, the signal is observed in the cytoplasm in the form of small granules. Both markers of Leydig cells significantly facilitated the recognition of these cells during morphometric (stereological) analysis.

**Figure 8:**
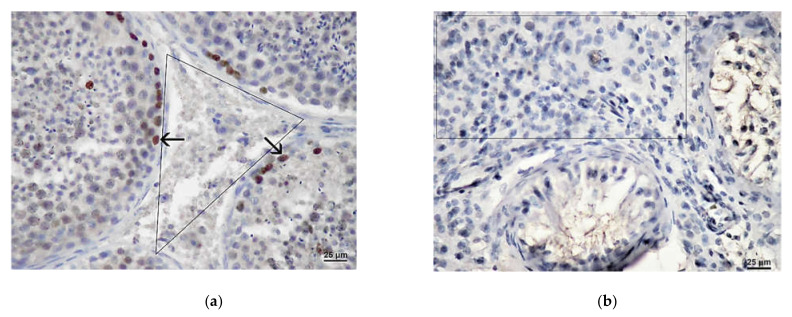
(**a**) Expression of Ki-67 (→) in the testis with maintained spermatogenesis. The expression was positive within spermatogenic cells, predominately spermatogonia. The interstitial area with Leydig cells (triangle) demonstrated no positivity. (DAB, ×400, scale = 25 µm); (**b**) Testicular biopsy from an infertile man with NOA, expression of Ki-67. Seminiferous tubules showed a Sertoli cell-only pattern. Within the interstitium, a large cluster of Leydig cells (rectangle) displayed no positivity. (DAB, ×400, scale = 25 µm).

In patients with OA, a marked presence of Ki-67 protein was demonstrated within the seminiferous tubules, especially in spermatogonia. The associated interstitium was negative (Figure 8a). In the group of patients with NOA and maturation arrest (relatively sustained spermatogenesis), Ki-67 expression was also most pronounced in the basal section of the seminal epithelium, within the spermatogonia nucleus. However, in severe cases of NOA (Sertoli cell-only syndrome, tubular fibrosis), the expression of the above protein was absent (Figure 8b). Ki-67 expression could also not be demonstrated in the interstitium of patients with NOA. This was especially true for smaller and/or larger groups of Leydig cells that were not Ki-67 positive (Figure 8b).

**Figure 9:**
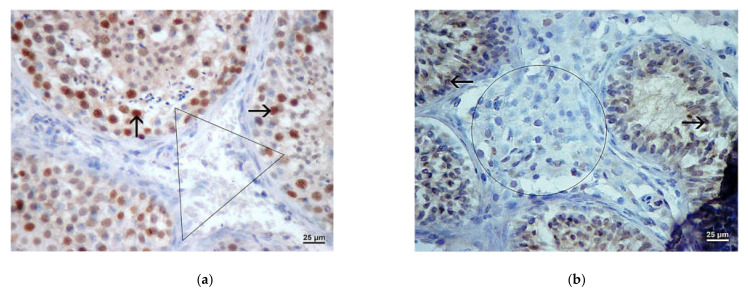
(**a**) Expression of PCNA (→) in a control testicular biopsy (obstructive azoospermia case). Within seminiferous tubules, a lot of spermatogenic cells were positive. Interstitium with Leydig cells (triangle) was negative. (DAB, ×400, scale = 25 µm); (**b**) Testicular biopsy from a patient with maturation arrest, expression of PCNA. However, some cells of seminiferous epithelium showed a proliferative activity (→), Leydig cells (circled area) were negative. (DAB, ×400, scale = 25 µm).

Almost the same result has been obtained applying antibody to proliferative cell nuclear antigen (PCNA). In the OA biopsies, abundant expression of PCNA was found in many spermatogenic cells undergoing meiosis/mitosis. However, no positivity was detected within Leydig cells of the interstitium (Figure 9a). In NOA biopsies, PCNA signal was reduced, depending on the degree of depletion of spermatogenic cells. Inside the tubules, only a few remaining spermatogenic cells were positive. The interstitium of NOA patients was devoid of PCNA signal (Figure 9b).

### 3.4. Results of Morphometric (Stereological) Analysis

Processing data obtained on testis samples from the OA group and men with NOA showed no normal distribution of data, which is why non-parametric tests (Mann–Whitney, Kruskal–Wallis) were used. The Kruskal–Wallis (Dunn’s post hoc) test showed no statistically significant difference between the investigated groups of patients. Comparison of the numerical density (NvLc, number of Leydig cells in a unit tissue volume) of the left and right testes by the Mann–Whitney test did not prove a statistically significant difference between the examined groups (*p* = 0.79 and *p* = 0.49) (Table 2). By comparing the volume density (VvLc, volume of Leydig cells in a unit tissue volume) of the left and right testes, a statistically significant difference was obtained between the OA and the group of patients with NOA (*p* ˂ 0.001 and *p* = 0.0014) (Table 3). The volume density of Leydig cells in the biopsies of patients with NOA was significantly higher than in the OA group.

The total number of Leydig cells (NLc) by the Mann–Whitney test showed a statistically significant difference between the OA group and patients with NOA (*p* = 0.0026 for the left testis and *p* = 0.0138 for the right testis). The total number of Leydig cells in the testes of patients with NOA was lower (Table 4). Analysis of the total volume of Leydig cells (VLc) in the testes of the examined groups of patients showed no statistically significant difference (*p* = 0.199 for the left and *p* = 0.131 for the right testis) (Table 5).

## 4. Discussion

The results of this study showed a completely normal histological picture and arrangement of spermatogenic and interstitial Leydig cells in the OA group. In almost all biopsy samples well-defined stages of spermatogenesis, characteristic of the spermatogenic epithelium cycle, were observed [13,15,24]. The loose interstitial connective tissue between the preserved seminiferous tubules bore typical clusters of peritubular and perivascular Leydig cells. Interstitial Leydig cells could be seen within the connective tissue in groups of 5–25 cells corresponding to their usual picture [14,24,25].

In the biopsies of the infertile group of men with NOA, various degrees of spermatogenesis damage were noted due to extensive re-modelling of the testicular parenchyma. The tubules showed a diversity of histological pictures, ranging from maturation arrest to a picture of “Sertoli cell-only” syndrome or tubular fibrosis (entirely fibrosed tubules or “tubular shadows”). In the case of Sertoli cells only syndrome, seminiferous tubules consisted only of Sertoli (supporting) cells. Such Sertoli cells often had extremely large vacuoles in their cytoplasm due to the loss of spermatogenic cells, corresponding to changes in these cells described in the literature [15,25]. The basal membrane of the seminal epithelium was sometimes thickened and lamina propria accumulated hyaline material (hyalinisation of the wall of the seminiferous tubules). The changes described above correspond to characteristics of the testicular parenchyma in patients with NOA reported in the literature [7,8,14,15].

In the testicular biopsy samples of patients with NOA, the interstitium contained Leydig cells of diverse morphology, ranging from regular clusters of Leydig cells, significantly hypertrophic groups of Leydig cells to a depletion of these cells due to interstitial fibrosis. Interstitial fibrosis was apparent in several NOA cases, with few Leydig cells remaining in the interstitium. These cells often had elongated cytoplasms and were surrounded by rich connective tissue. The areas of interstitial fibrosis bore abundant presence of fibroblasts, fibrocytes, and connective tissue ground substance. Moreover, the interstitial fibrosis was frequently accompanied by inflammatory cells, i.e., mononuclear infiltrations. These infiltrations were more often of a peritubular arrangement. Sometimes mononuclear cells invaded the lamina propria of seminiferous tubules, not only a connective tissue around the tubules. Another pattern of arrangement of inflammatory cells was typically around the blood vessels of the interstitium, affecting perivascular Leydig cells in their vicinity. Sometimes the histological picture of mononuclear infiltrates was mixed, with both peritubular and perivascular arrangement.

The results of the immunohistochemical part of this study indicated marked differences between the OA and patients with NOA. In patients with OA, a considerable presence of Ki-67 cell proliferation marker within the seminiferous tubules/spermatogenic cells was demonstrated. This was especially true for spermatogonia. The associated interstitium with Leydig cells was negative. However, in severe cases of NOA (Sertoli cell-only syndrome, tubular fibrosis), the expression of this marker was absent in the tubules as well. The Ki-67 expression could also not be demonstrated in the interstitium of patients with NOA, despite the occasional presence of large groups/clusters of Leydig cells. The expression of PCNA in investigated groups of patients followed the pattern of expression described for Ki-67. Again, there was no PCNA expression in the interstitial compartment with Leydig cells.

The results of morphometric (stereological) analysis showed that the volume density of Leydig cells (VvLc) in biopsies of patients with NOA was significantly higher than in the OA group. This variable reflects histological observations described in our study: the existence of large and hypertrophic clusters of Leydig cells, especially in severe cases of NOA, such as Sertoli cells only syndrome and/or tubular fibrosis. The numerical density of Leydig cells (NvLc) in the NOA group was not significantly increased. However, volume and numerical density are relative stereological variables that reflect changes in a unit tissue volume rather than whole organ [22,23].

The analysis of the total number (NLc) and volume (VLc) of Leydig cells in the whole organ showed no normal data distribution. The total Leydig cell number analysis showed a statistically significant difference between the control group and patients with NOA. The total number of Leydig cells in the testes of patients with NOA was statistically significantly lower than in the OA group. However, the total volume of Leydig cells in NOA patients was not significantly lower compared to the OA group.

Most reproductive studies point to the fact that 15% of couples of regenerative age are infertile. In 50% of cases, the cause of infertility is a “male factor”. The origins of male infertility are various; however, sometimes, the cause cannot be detected even after detailed processing. It has been proven that infections and inflammatory processes are responsible for developing male infertility [26,27,28]. Sexually transmitted diseases such as Chlamydia trachomatis, Ureaplasma, *E. coli*, etc., can cause ascending infection of the epididymis and testicles. The infection leads to several inflammatory responses that can cause the deterioration of the seminal epithelium and the consequent formation of fibrosis. In addition to sexually transmitted diseases, infection and the inflammatory response can also be caused by some systemic viral inflammations that by hematogenous spread can cause infection and inflammation in the testes [28,29,30]. Pathogens themselves, their components or inflammatory factors can cause irreparable damage to the testicles and epididymis.

Several studies have investigated the influence of inflammatory factors on the development of testicular interstitial fibrosis and the deterioration of the seminiferous epithelium. The results showed that the levels of activin A and key fibrogenic proteins increased in human testicular biopsies with leukocyte infiltrates and impaired spermatogenesis. The same goes for autoimmune orchitis (EAO) induced in the mouse [31]. EAO is characterised by the production of testicular auto-antibodies, elevated levels of inflammatory mediators, including tumour necrosis factor (TNF) and activin A. In doing so, activin A stimulates the formation of fibrose tissue around peritubular cells. Moreover, there is a leukocyte interstitial infiltration, severe seminiferous tubules damage and apoptosis of spermatogenic cells resembling histopathological disorders in infertile men [26,31,32,33].

Activin A is a key regulator of fibrosis and inflammation in various tissues, highlighting its potential as a target molecule in diagnosis and treatment. It regulates fibrosis by stimulating fibroblast proliferation and differentiation into myofibroblasts. Stimulated myofibroblasts are key cells that produce excessive amounts of type I and III collagen and fibronectin, leading to fibrosis formation. Pathohistological analysis of testicular biopsies obtained in asymptomatic infertile men showed marked interstitial infiltration by immune cells in approximately 30% of cases, coinciding with seminal epithelial loss, lamina propria thickening, and tubular fibrosis [27,28,34,35].

Data on the nature of human Leydig cells within the adult testis indicate three types/subpopulations of these cells can be distinguished by their specific markers: progenitor, immature and mature Leydig cells [36]. New data on single-cell analysis confirmed the existence of these three types [37]. It seems that in the normal testis with regular spermatogenesis, there is a certain balance between Leydig cell subpopulations, with the predominance of mature Leydig cells. However, in the case of Klinefelter’s syndrome, characterised by huge accumulations of Leydig cells, there is a growth of the progenitor and immature cell types [36]. Our immunohistochemistry data indicated no proliferative activity of Leydig cells in both investigated groups of patients (OA, NOA). However, since we observed large clusters of Leydig cells in some NOA cases, one can speculate on increased differentiation of progenitor and immature Leydig cells. These cells would not necessarily proliferate, i.e., undergo cell hyperplasia, but could change their morphology towards more differentiated (mature) Leydig cells. Moreover, they could probably acquire INSL-3 expression as well as testosterone production.

Our stereological data indicate a lower total number and volume of Leydig cells in the NOA group compared to OA. During the inspection of histological specimens, we have noticed in a significant number of biopsies tubules with Sertoli cells only syndrome and tubular fibrosis. The changes of seminiferous tubules were frequently accompanied by the marked fibrosis of the interstitial compartment, the presence of mononuclear infiltrations and depletion of Leydig cells. Thus, since our study is based on a single-centre experience, it bears, as such, clear limitations. As a tertiary andrological centre, we deal with the most difficult cases of infertility. Almost 90% of patients with azoospermia in our centre are NOA cases [38], where parenchyma re-modelling and fibrosis is profound. 

Given that NOA is characterised by intense re-modelling of the testicular parenchyma, where inflammation and fibrosis play an important role, the decrease in the number and volume of Leydig cells might not be surprising. Compared with previous studies [13], the present study included more cases, and a more comprehensive stereological analysis was performed. Future studies should deal with more homogeneous subpopulations of NOA patients to clarify whether Leydig cells undergo actual hyperplasia. Ideally, it should be a multi-centre study with a large number of patients.

## 5. Conclusions

Our study analysed the possible proliferative activity of Leydig cells in NOA patients. Leydig cells were evaluated by qualitative histological analysis on H&E slides, immunohistochemistry, and stereology. The stereological analysis results showed no increase in the number of Leydig cells; on the contrary, the comparison of the examined groups of patients showed a decrease in their number in the biopsy samples of patients with NOA. This decrease in Leydig cells can be explained by previous and simultaneous inflammatory changes within the testicular interstitium that caused consequent interstitial fibrosis. The interstitial fibrosis could induce an impairment of function and, finally, depletion of Leydig cells.

## Figures and Tables

**Table 1:** Hormone values in investigated groups of patients.

Group	FSH (IU/L) Mean Range (min.–max.)	LH (IU/L) Mean Range (min.–max.)	T (nmol/L) Mean Range (min.–max.)
OA	4.36	3.59	19.8
	(1.9–6.7)	(1.2–6.8)	(12.4–28.6)
NOA	29.18	13.24	17.63
	(2.8–41.9)	(1.7–23.7)	(7.7–39.2)

FSH, follicle stimulating hormone (normal values: 2–10 IU/L); LH, luteinizing hormone (normal values: 1–8 UI/L); T, total testosterone (normal values: (10–35 nmol/L).

**Table 2:** Numerical density of Leydig cells in testes of investigated groups of patients.

	N_V_(mm^−2^)/Leydig Cells			
	OA Left Testis	OA Right Testis	NOA Left Testis	NOA Right Testis
Mean	6243	6225	7188	7508
SD	3927	4533	5672	6602
SE	742.2	906.6	987.3	1167

Mann–Whitney test; SD (Standard Deviation); SE (Standard Error); *p* > 0.05.

**Table 3:** Volume density of Leydig cells in testes of investigated groups of patients.

	V_V_(mm^0^)/Leydig Cells			
	OA Left Testis	OA Right Testis	NOA Left Testis	NOA Right Testis
Mean	0.03857	0.0384	0.08030 ^a^	0.08281 ^b^
SD	0.01957	0.01818	0.08278	0.08865
SE	0.003699	0.003637	0.01441	0.01567

Mann–Whitney test; SD (Standard Deviation); SE (Standard Error); ^a^ *p* = 0.0001; ^b^ *p* = 0.0014.

**Table 4:** Total number of Leydig cells in testes of investigated groups of patients.

	N × 10^6^/Leydig Cells			
	OA Left Testis	OA Right Testis	NOA Left Testis	NOA Right Testis
Mean	138.9	120.3	45.96 ^a^	34.41 ^b^
SD	81.44	67.18	38.39	16.66
SE	18.68	16.29	14.51	8.331

Mann–Whitney test; SD (Standard Deviation); SE (Standard Error); ^a^ *p* = 0.0026; ^b^ *p* = 0.0138.

**Table 5:** Total volume of Leydig cells in testes of investigated groups of patients.

	V/mm^3^/Leydig Cells			
	OA Left Testis	OA Right Testis	NOA Left Testis	NOA Right Testis
Mean	768.7	676.0	490.1	402.5
SD	490.6	302.9	349.6	204.1
SE	112.6	73.46	132.1	102.0

Mann–Whitney test; SD (Standard Deviation); SE (Standard Error); *p* > 0.05.

## Data Availability

Not applicable.
